# SMBE Secretary’s Report

**DOI:** 10.1093/molbev/msae104

**Published:** 2024-05-31

**Authors:** Emmanuelle Lerat

**Affiliations:** Universite Claude Bernard Lyon 1, LBBE, UMR 5558, CNRS, VAS, Villeurbanne F-69622, France

## Council Meetings

### I. Call to Order

President Kateryna Makova called the council meeting to order at 8:30 AM Central European Summer Time (CEST) on July 23, 2023, in the presence of President Kateryna Makova, Past President James McInerney, President-Elect Stephen Wright, Councilors Josefa Gonzalez, Katerina Gushanski, Maria Avila Arcos, Christian Landry, and Rebecca Zufall, Secretary Emmanuelle Lerat, MBE Editors-in-Chief Brandon Gaut (*ex officio*) and Claudia Russo (*ex officio*), and GBE Editors-in-Chief Laura A. Katz (*ex officio*) and Adam Eyre-Walker (*ex officio*). In electronic attendance were Treasurer John McCutcheon and Councilor Jeffrey Ross-Ibarra.

#### Noncouncil Members in Attendance

Lulu Stader (Executive Administrator), Rodhri Jackson (publishers OUP, MBE, and GBE), and Chris Lapine (Allen Press) also attended.

### II. Treasurer’s Report

Treasurer John McCutcheon presented the annual Treasurer’s report, with details on journal and meeting revenue and expenses. The society continues to be financially healthy. The Council discussed several priorities moving forward to reduce our net assets and best serve our membership. The four EiCs and the Treasurer suggest that the Council forms a subcommittee to work toward ensuring the long-term financial stability of the society. Full details can be found in the complete Treasurer’s report.

### III. Secretary’s Report

Secretary Emmanuelle Lerat briefly reviewed the Council’s actions over the preceding year. The Council voted on eight motions between January 1 and July 23, 2023. The Council approved (i) the funding of two regional and three satellite meetings, (ii) the choice of an option for the code of conduct revision, (iii) increasing the reimbursement amount for council members and AEs to attend the SMBE meeting, (iv) to reimburse noneconomy travel due to medical condition, (v) the appointment of new AEs for GBE, (vi) increasing the number of travel awards to travel to SMBE2023, (vii) GBE special edition based on SMBE 2023 symposia, and (viii) the decision about a potential violation of our code of conduct. Full details can be found in the complete Secretary’s report.

### IV. Election of SMBE Officers

A nominations committee to nominate candidates for the position of President-elect, Treasurer, and two councilor positions was chaired by Aoife McLysaght, Deepa Agashe, Sandra Baldauf, Rosa María Fernández, Barbara Holland, Yuseob Kim, Wojciech Makalowski, Juan C. Opazo, and Emmanuelle Lerat (ex officio, SMBE Secretary, as per by-laws). The membership of the society was informed by email of the committee composition in early 2023 and asked to nominate candidates for the positions. The Council does and will continue to remind the nominating committee every year to prioritize gender and geographic representation among the candidates, but it maintains flexibility in how this is achieved by the committee.

The final selected nominees were Mar Alba and Ziheng Yang for President-elect, Aline Muyle and Julian Echave for Treasurer, and Elena Gómez-Díaz, Alexander Suh, Koichiro Tamura, and Juliana Vianna for Councilor. The membership of the society was issued online ballot details through Allen Press. In total, 487 members voted in this election out of 2,995 members invited. This corresponds to a 16% participation rate, which is higher than the previous year but not as high as the highest historical rates. The results of the election are that President-elect is Ziheng Yang, Treasurer is Aline Muyle, and Councilors are Elena Gómez-Díaz and Juliana Vianna. The full nominations report is available as a separate document.

### V. Editors’/OUP/AP Reports

The Editors-in-Chief of MBE and GBE gave reports on the current states of their journals (see separate editors’ reports for full details). For GBE, 2022 to 2023 was a tough year as the submission number declined more than other journals and the impact factor (IF) dropped this year. The reasons for these declines are not obvious and continue to be investigated with OUP. The infrastructure to support the MBE–GBE transfer process is efficient and operating smoothly. Two undergraduate students have been appointed to check for data availability statements. MBE continues to be in a very strong position although with a reduction in IF in 2022 that increases again in 2023. After a record number of submissions in 2021, there is a decrease since then that could still be the impact of covid but also to the arrival of new journals in the field.

OUP proposes MBE and GBE to join Straive’s Transfer Desk Initiative Pilot. It was decided by the council for a test for 6 months with a feedback at that time.

### VI. Other Activities

#### Future Annual Meetings

SMBE 2024 will be in Puerto Vallarta, Mexico. SMBE 2025 will be in Beijing, China. It was decided that the two meetings should be hybrid as the SMBE 2023 meeting to allow better accessibility. We have solicited proposals for SMBE 2026 from Europe.

#### IDEA Task Force, Proposals

The call for IDEA proposals resulted in the funding of three proposals: (i) Virtual Lab Meeting Training Program (proposed by Joanna Kelley), (ii) applying feminist/antioppressive lenses to evolutionary genetics (proposed by Emily Josephs, Daniela Palmer, and K. Supriya), and (iii) EvoBio Crash Course: Bridging the Knowledge Gap (proposed by Sergio González et al.).

An IDEA symposium was organized during the SMBE 2023 meeting.

#### Career Awards

Forty-five people were supported by this award to attend the SMBE 2023 meeting in Ferrara, Italy.

#### Early Career Award

This award is given to outstanding members of the SMBE community who are in the early stages of an independent research career. The 2023 recipient was Amy Goldberg at Duke University.

#### Mid-career Award

This award is intended for outstanding members of the SMBE community who are in the midst of their research careers. The 2023 recipient was Kayla King at the University of British Columbia.

#### Lifetime Achievement Award

This award is intended for outstanding senior members of the SMBE community. The 2023 recipient was Jim Lake from UCLA.

#### Service Award

This award is intended for individuals who have provided extraordinary service to SMBE community. The 2023 recipients were Beth Shapiro and Mary O’Connell.

#### Graduate Student Excellence Awards

This Symposium provides a forum for young investigators to showcase their exemplary research and continues to be a highlight of the SMBE annual meeting. We celebrate all of the candidates in this Symposium. The awardees were as follows:

Kai Yan (Kunming Institute of Zoology, China)
Carmina Barberena-Jonas (Unidad de Genómica Avanzada [UGA], Mexico)
Matteo Sebastianelli (University of Cyprus, Cyprus)
Aurora Alvarez-Buylla (Stanford University, USA)
Harvinder Pawar (Institute of Evolutionary Biology [UPF-CSIC])
Ujani Hazra (Georgia Tech, USA)
Carlos R. Cortez-Romero (University of Chicago, USA)
Margaux Aubel (University of Münster, Germany)
Marianne Dehasque (Stockholm University/Swedish Museum of Natural History, Sweden)

#### Best Graduate Student Paper Award

These awards provide recognition for outstanding student papers in both SMBE journals. For MBE, the committee selected one winner.

*Winner*: Reena Debray: “Historical Contingency Drives Compensatory Evolution and Rare Reversal of Phage Resistance”
For GBE, the committee selected one winner.
*Winner*: Rebekka Müller: “A Nearly Neutral Model of Molecular Signatures of Natural Selection after Change in Population Size”

#### SMBE Undergraduate Mentorship Awards 2023

These awards were granted to 19 undergraduate students to attend the SMBE 2023 meeting in Ferrara, Italy. In this program, each student is paired with a mentor to assist them throughout the SMBE meeting.

### VII. Additional Business

Council discussed revisiting the current and proposed future OUP contract.

## Business Meeting

This year’s business meeting was conducted on July 26, 2023, during the SMBE meeting in Ferrara, Italy. The President presented a financial overview of the society. All of the awards were highlighted. The President presented an overview of the membership categories and a membership update and also reviewed the benefits of the membership. She provided some statistics about our journals, including impact factors and submission rates. The President reported the election results and the locations of SMBE 2024 and 2025. She also presented the upcoming regional and satellite meetings. The work of the IDEA taskforce was highlighted.

